# Supplementary material for: Self-renewing human naïve pluripotent stem cells dedifferentiate in 3D culture and form blastoids spontaneously
Source: Nat Commun. 2024 Jan 22;15:668. doi: 10.1038/s41467-024-44969-x (PMC10803796; doi:10.1038/s41467-024-44969-x)
Supplement: Supplementary file 1 — Supplementary Information [file 41467_2024_44969_MOESM1_ESM.pdf]

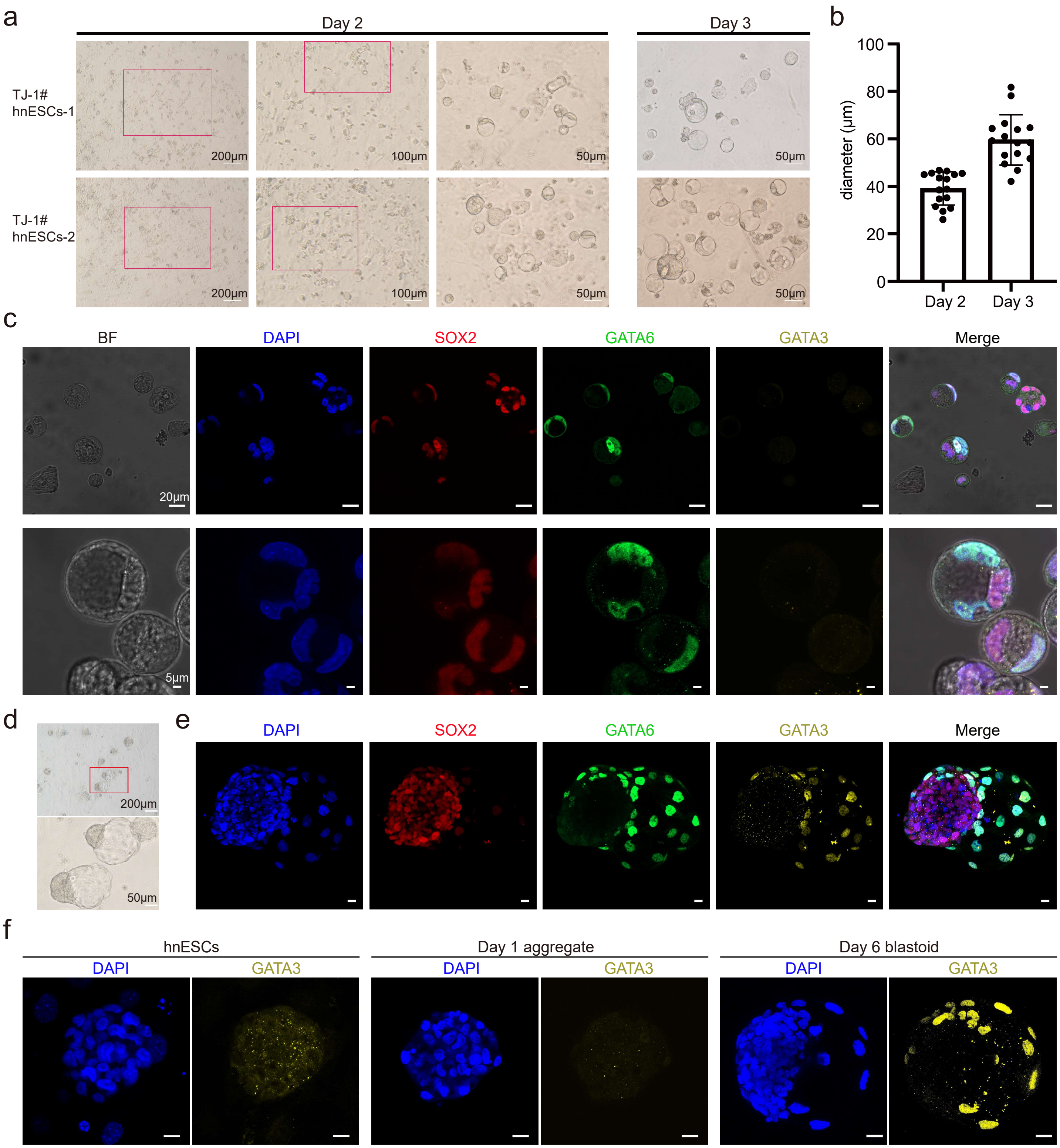

Supplementary Figure 1. **Blastocyst-like structures emerge spontaneously during hnESC culture.** **a** Representative phase-contrast images of spontaneous cysts in the hnESCs cultures under different magnifications. **b** Quantification of diameter of spontaneous cysts (day2, n = 16, 39.13±6.95μm; day3, n = 16, 59.61±10.55μm). **c** Representative immunofluorescence staining images of spontaneous cysts in (a). **d** Representative phase-contrast images of blastocyst-like structures from day 6 suspended hnESC colonies. **e** Representative immunofluorescence staining images of blastocyst-like structures in (d). Scale bars, 20μm. **f** Representative immunofluorescence staining images of hnESCs, day 1 aggregates and day 6 blastoids from AggreWell . Scale bars, 20μm.

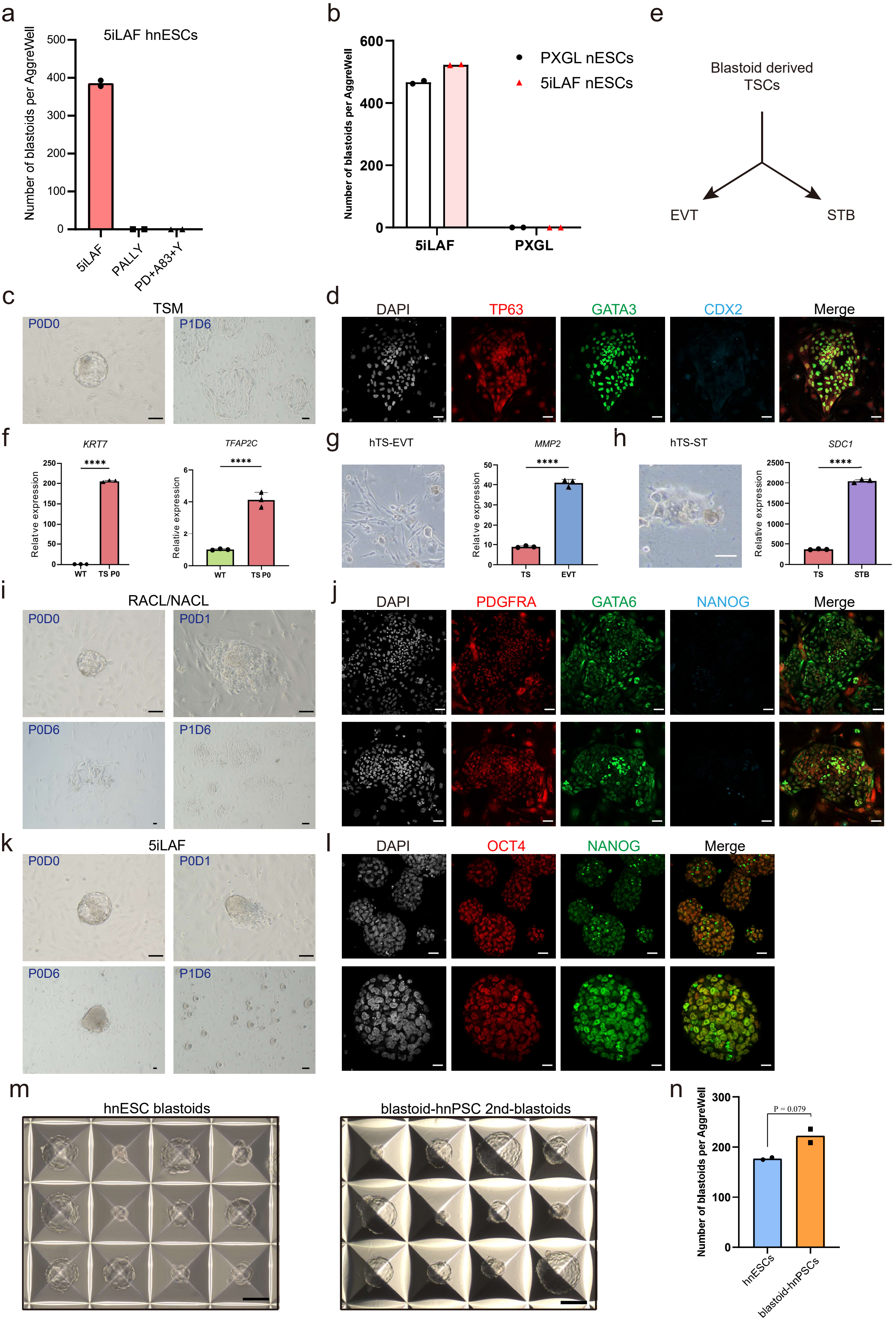
a
5iLAF hnESCs
Number of blastoids per AggreWell
500
400
300
200
100
0
5iLAF
PALLY
PD+A83+Y
b
600
400
200
0
Number of blastoids per AggreWell
PXGL nESCs
5iLAF nESCs
5iLAF
PXGL
e
Blastoid derived
TSCs
EVT
STB
c
TSM
P0D0
P1D6
d
DAPI
TP63
GATA3
CDX2
Merge
f
KRT7
TFAP2C
Relative expression
****
WT
TS P0
g
hTS-EVT
MMP2
TS
EVT
h
hTS-ST
SDC1
TS
STB
i
RACL/NACL
P0D0
P0D1
P0D6
P1D6
j
DAPI
PDGFRA
GATA6
NANOG
Merge
k
5iLAF
P0D0
P0D1
P0D6
P1D6
l
DAPI
OCT4
NANOG
Merge
m
hnESC blastoids
blastoid-hnPSC 2nd-blastoids
n
Number of blastoids per AggreWell
300
200
100
0
P = 0.079
hnESCs
blastoid-hnPSCs

Supplementary Figure 2. **Generation of spontaneous blastoids and derivation of early embryo-like stem cell lines. a** Number of blastoids generated from hnESCs in AggreWells with 5iLAF medium or published protocols. PALLY; PD+A83+Y. **b** Number of blastoids generated from PXGL and 5iLAF cultured hnESCs in AggreWells with 5iLAF or PXGL media. **c, d** Derivation (c) and representative immunofluorescence staining images (d) of TSCs from day6-blastoids. TSM, trophectoderm stem cell medium. P0D0, day 0 of passage 0 and so on. Scale bars, 100μm. **e** Schematic of EVT and ST differentiation from blastoids-derived TSCs. **f** Quantitative gene expression of selected TS markers in P0 TSCs. **g, h** Brightfield images and representative marker expression of EVT like cells (g) and ST like cells (h) differentiated from spontaneous blastoid-derived TSCs respectively. Scale bars, 50μm. **i, j** Derivation (i, scale bars, 100μm) and representative immunofluorescence staining images (j, scale bars, 50μm) of nEnd cells from day6-blastoids. RACL and NACL, nEnd cells derivation and culture media, respectively. P1D6, day6 of passage 1 and so on. **k, l** Derivation (k, scale bars, 100μm) and representative immunofluorescence staining images (l, scale bars, 50μm) of blastoids-nPSCs from day6-blastoids. **m, n** Representative phase-contrast images (m) and quantification (n) of blastoids generated from hnESCs or blastoids-nPSCs in AggreWell with 5iLAF medium. Scale bars, 200μm. Unpaired two-tailed t-test. WT: hnESCs, TS P0: 1st generation of trophoblast stem cells directly derived from blastoids, TS: maintained blastoid-derived trophoblast stem cell line, EVT: extravillous trophoblast like cells, ST: syncytiotrophoblast like cells.

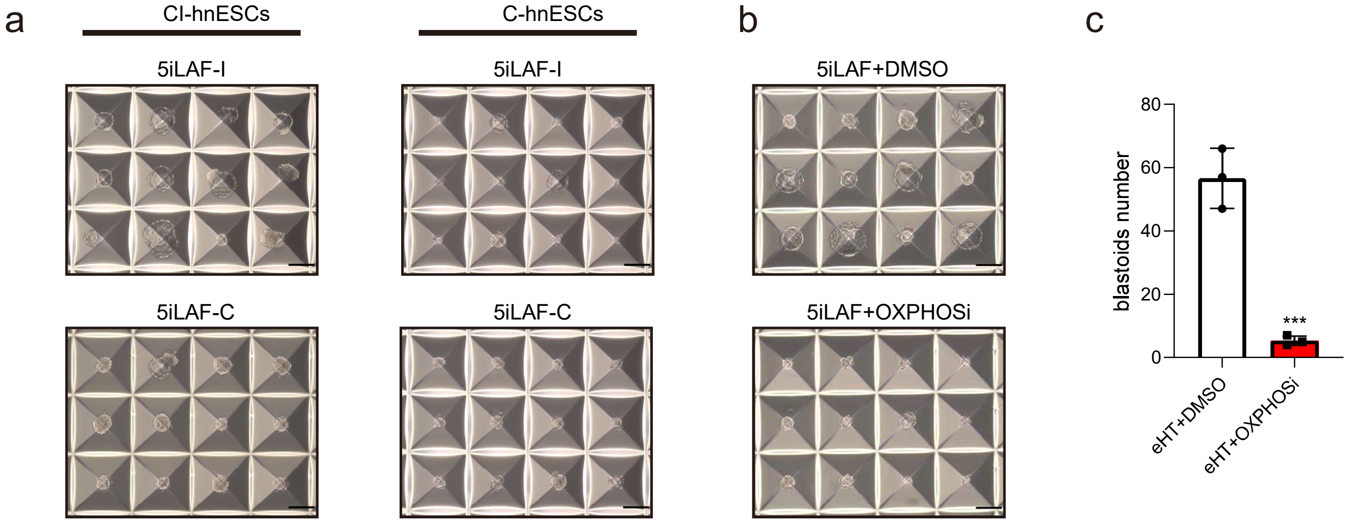

Supplementary Figure 3. **OXPHOS underlies the ability of hnESCs to generate blastoids spontaneously. a** Representative phase-contrast images of blastoids generated from CI-hnESCs (left) and C-hnESCs (right) with 5iLAF-I or 5iLAF-C medium in AggreWells. Scale bars, 200μm. **b** Representative phase-contrast images of blastoids generated from CI-hnESCs in 5iLAF-I with DMSO or OXPHOS inhibitor (Fig. 2h). **c** Blastoid inducing efficiency in the presence of DMSO or OXPHOS inhibitor (IACS-010759, 5μM) following reported eHT protocol.

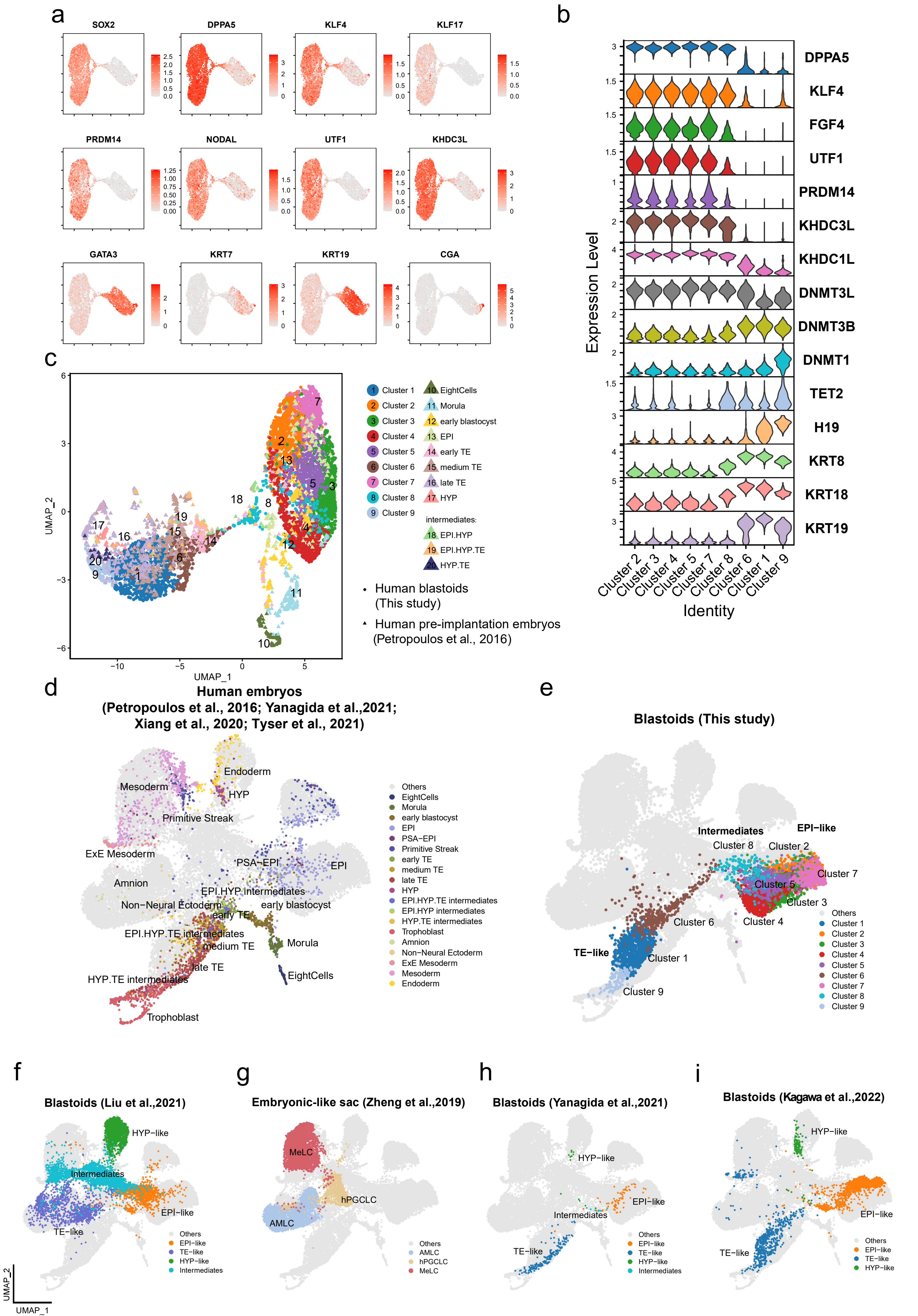

a
SOX2
DPPA5
KLF4
KLF17
PRDM14
NODAL
UTF1
KHDC3L
GATA3
KRT7
KRT19
CGA
b
DPPA5
KLF4
FGF4
UTF1
PRDM14
KHDC3L
KHDC1L
DNMT3L
DNMT3B
DNMT1
TET2
H19
KRT8
KRT18
KRT19
Expression Level
Cluster 2
Cluster 3
Cluster 4
Cluster 5
Cluster 7
Cluster 8
Cluster 6
Cluster 1
Cluster 9
Identity
c
UMAP_2
UMAP_1
1 Cluster 1
2 Cluster 2
3 Cluster 3
4 Cluster 4
5 Cluster 5
6 Cluster 6
7 Cluster 7
8 Cluster 8
9 Cluster 9
10 EightCells
11 Morula
12 early blastocyst
13 EPI
14 early TE
15 medium TE
16 late TE
17 HYP
intermediates:
18 EPI.HYP
19 EPI.HYP.TE
20 HYP.TE
Human blastoids (This study)
Human pre-implantation embryos (Petropoulos et al., 2016)
d
Human embryos (Petropoulos et al., 2016; Yanagida et al.,2021; Xiang et al., 2020; Tyser et al., 2021)
Endoderm
Mesoderm
HYP
Primitive Streak
ExE Mesoderm
PSA-EPI
EPI
Amnion
EPI.HYP intermediates
Non-Neural Ectoderm
early blastocyst
early TE
EPI.HYP.TE intermediates
Morula
medium TE
late TE
EightCells
HYP.TE intermediates
Trophoblast
Others
EightCells
Morula
early blastocyst
EPI
PSA-EPI
Primitive Streak
early TE
medium TE
late TE
HYP
EPI.HYP.TE intermediates
EPI.HYP intermediates
HYP.TE intermediates
Trophoblast
Amnion
Non-Neural Ectoderm
ExE Mesoderm
Mesoderm
Endoderm
e
Blastoids (This study)
Intermediates
EPI-like
Cluster 8
Cluster 2
Cluster 7
Cluster 5
Cluster 3
Cluster 6
Cluster 4
TE-like
Cluster 1
Cluster 9
Others
Cluster 1
Cluster 2
Cluster 3
Cluster 4
Cluster 5
Cluster 6
Cluster 7
Cluster 8
Cluster 9
f
Blastoids (Liu et al.,2021)
HYP-like
Intermediates
EPI-like
TE-like
Others
EPI-like
TE-like
HYP-like
Intermediates
UMAP_2
UMAP_1
g
Embryonic-like sac (Zheng et al.,2019)
MeLC
hPGCLC
AMLC
Others
AMLC
hPGCLC
MeLC
h
Blastoids (Yanagida et al.,2021)
HYP-like
EPI-like
Intermediates
TE-like
Others
EPI-like
TE-like
HYP-like
Intermediates
i
Blastoids (Kagawa et al.,2022)
HYP-like
EPI-like
TE-like
Others
EPI-like
TE-like
HYP-like

Supplementary Figure 4. **scRNA-seq analyses of human spontaneous blastoids.** **a** Expression levels of human blastocyst lineage markers. **b** Expression levels of representative genes enriched in clusters. **c** Joint UMAP of single-cell transcriptomes of cells from day6-blastoids (Fig.3f) integrated with published datasets from human early embryos (Fig.3e). **d-i** Joint UMAP of single-cell transcriptomes of cells from day6-blastoids (e) integrated with published datasets (d, f-i). PSA-EPI, primitive streak anlage stage epiblast. ExE, extraembryonic. AMLC, amniotic ectoderm-like cells. hPGCLC, human primordial germ cells -like cells. MeLC, Mesoderm-like cell.

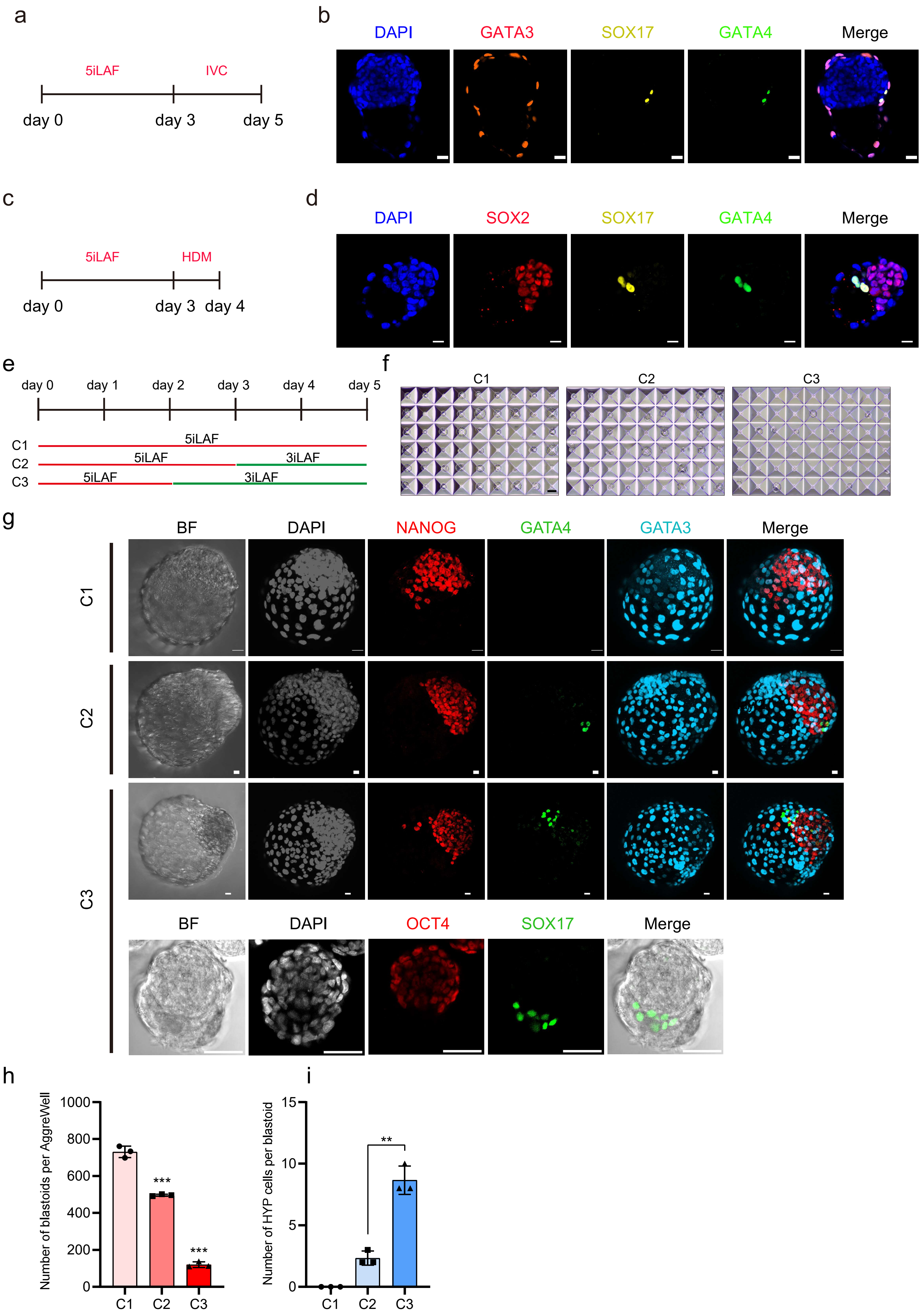
a
5iLAF
IVC
day 0
day 3
day 5
b
DAPI
GATA3
SOX17
GATA4
Merge
c
5iLAF
HDM
day 0
day 3
day 4
d
DAPI
SOX2
SOX17
GATA4
Merge
e
day 0
day 1
day 2
day 3
day 4
day 5
C1
5iLAF
C2
5iLAF
3iLAF
C3
5iLAF
3iLAF
f
C1
C2
C3
g
BF
DAPI
NANOG
GATA4
GATA3
Merge
C1
C2
C3
BF
DAPI
OCT4
SOX17
Merge
h
Number of blastoids per AggreWell
1000
800
600
400
200
0
***
***
C1
C2
C3
i
Number of HYP cells per blastoid
15
10
5
0
**
C1
C2
C3

Supplementary Figure 5. **Spontaneous blastoids specify hypoblast-like cell fate. a-d** Schematic and representative immunofluorescence images of HYP-like cell containing blastoids generated using indicated culture conditions (a-d). Scale bars, 20μm. **e** Schematic of HYP-like cell induction by removing BRAF/MEK inhibitors. **f** Representative phase-contrast images showing blastoids in AggreWell. **g** Representative immunofluorescent images showing specification of GATA4+ or SOX17+ HYP-like cells in spontaneous blastoids. Scale bars, 20 μm for GATA4 staining and 100 μm for SOX17 staining. **h** Quantitative scoring of number of blastoids per AggreWell generated from the 3 different strategies. n = 3 (technical replicates). **i** Quantitative scoring of number of HYP cells per blastoid generated from the 3 different methods, n = 3 (technical replicates).

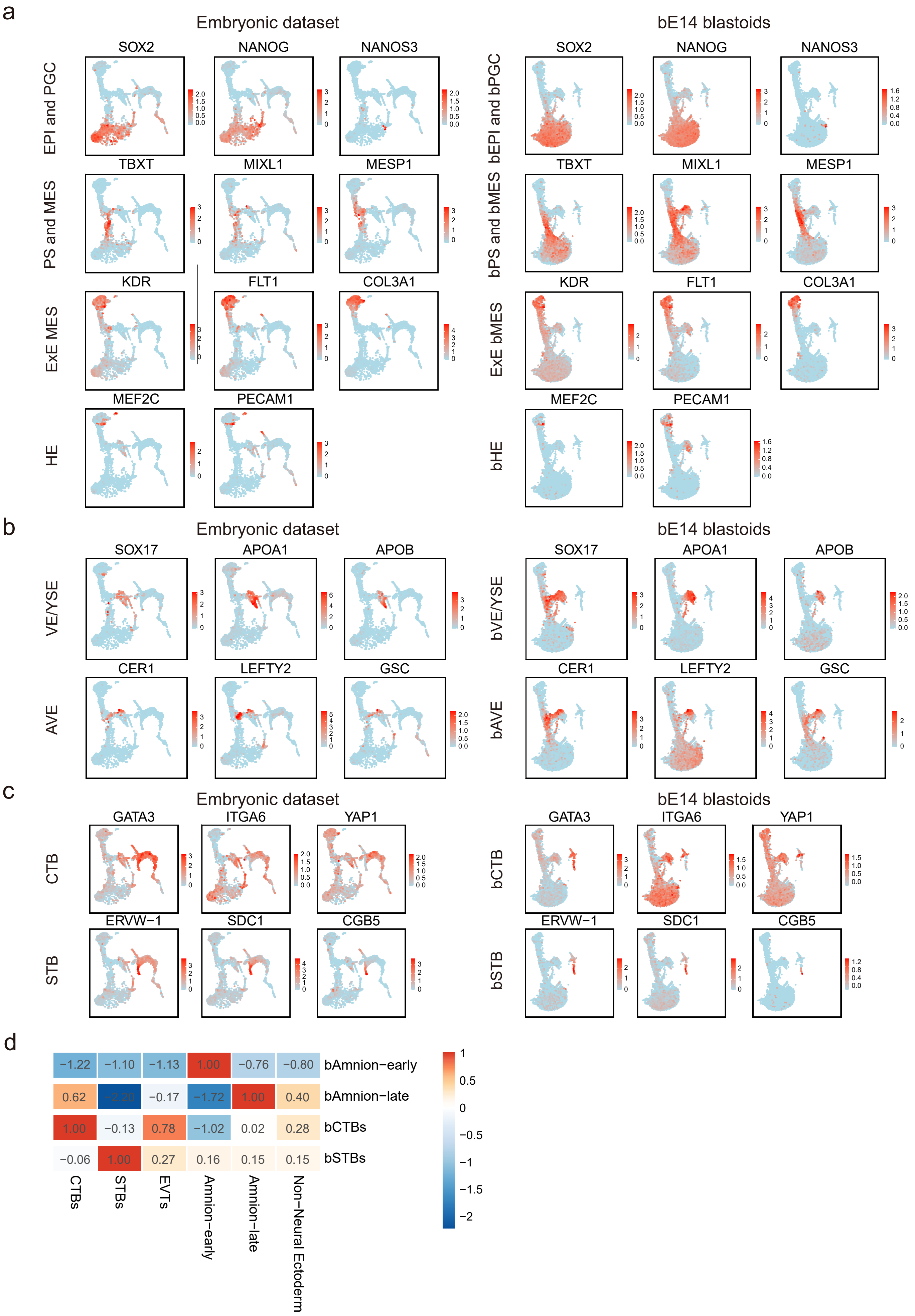
a
Embryonic dataset
bE14 blastoids
EPI and PGC
SOX2
NANOG
NANOS3
PS and MES
TBXT
MIXL1
MESP1
ExE MES
KDR
FLT1
COL3A1
HE
MEF2C
PECAM1
bEPI and bPGC
bPS and bMES
ExE bMES
bHE
b
Embryonic dataset
bE14 blastoids
VE/YSE
SOX17
APOA1
APOB
AVE
CER1
LEFTY2
GSC
bVE/YSE
bAVE
c
Embryonic dataset
bE14 blastoids
CTB
GATA3
ITGA6
YAP1
STB
ERVW−1
SDC1
CGB5
bCTB
bSTB
d
bAmnion−early
bAmnion−late
bCTBs
bSTBs
CTBs
STBs
EVTs
Amnion−early
Amnion−late
Non−Neural Ectoderm
−1.22 −1.10 −1.13 1.00 −0.76 −0.80
0.62 −2.20 −0.17 −1.72 1.00 0.40
1.00 −0.13 0.78 −1.02 0.02 0.28
−0.06 1.00 0.27 0.16 0.15 0.15
1
0.5
0
−0.5
−1
−1.5
−2

Supplementary Figure 6. **Spontaneous blastoids have post-implantation development potential.** **a** Featureplot displaying key lineage markers between embryonic dataset (left) and bE14 blastoids. **b** Featureplot displaying key extraembryonic endodermal lineage markers between embryonic dataset (left) and bE14 blastoids. **c** Featureplot displaying key extraembryonic trophoblast lineage markers between embryonic dataset (left) and bE14 blastoids. **d** Gene set variance analysis (GSVA) comparing similarities of clusters bAmnion-early and -late to natural embryo trophoblast lineages.

a

	C-hniPSC		CI-hniPSC	
	number of colonies picked	number of blastoids formed	number of colonies picked	number of blastoids formed
5iLAF-C	53	0	44	1
	76	0	48	1
	68	0	45	0
5iLAF-I	48	3	38	11
	68	5	43	10
	40	3	41	7

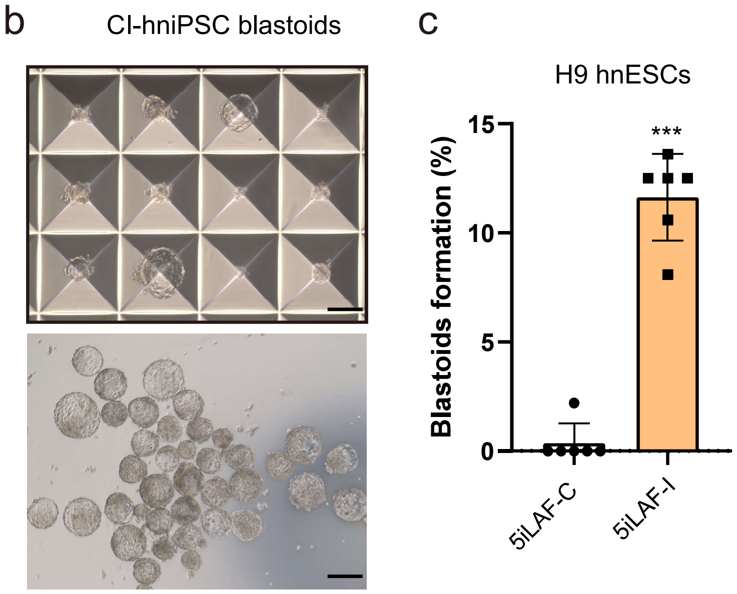

Supplementary Figure 7. **Spontaneous blastoid generation in independent human PSCs. a** A table showing the exact statistics of picked colonies and formed blastoids number from C-hniPSC or CI hniPSC in 5iLAF-C or 5iLAF-I media. **b** Representative phase-contrast images of human spontaneous blastoids generated from dissociated CI-hniPSCs in AggreWell. Scale bars, 200μm. **c** Blastoids formation efficiency of picked H9 CI-hnESC colonies in 5iLAF-C (n = 6 technical replicates) and in 5iLAF-I (n = 6) media. mean ± s.d.; unpaired two-tailed t-test; ***P < 0.001.
