# Supplementary material for: Self-renewing human naïve pluripotent stem cells dedifferentiate in 3D culture and form blastoids spontaneously
Source: Nat Commun. 2024 Jan 22;15:668. doi: 10.1038/s41467-024-44969-x (PMC10803796; doi:10.1038/s41467-024-44969-x)
Supplement: Supplementary file 3 — Description of Additional Supplementary Files [file 41467_2024_44969_MOESM3_ESM.pdf]

Description of Additional Supplementary Files

File Name: Supplementary Data 1
Description: Differentially expressed genes (padj < 0.05) are listed based on the comparison of CI-hnESCs and C-hnESCs.

File Name: Supplementary Data 2
Description: Top 50 enriched genes in each cluster of human day 6- blastoids.

File Name: Supplementary Data 3
Description: A list of antibodies used in this study.

File Name: Supplementary Data 4
Description: Lists of differentially expressed genes (padj < 0.05 and Log2FC > 1) used in Gene-Set Variation Analysis.

File Name: Supplementary Movie 1
Description: Live imaging of formation of spontaneous blastoids.

File Name: Supplementary Movie 2
Description: One-shot imaging of spontaneous blastoids in AggreWells.
