# Supplementary material for: Self-renewing human naïve pluripotent stem cells dedifferentiate in 3D culture and form blastoids spontaneously
Source: Nat Commun. 2024 Jan 22;15:668. doi: 10.1038/s41467-024-44969-x (PMC10803796; doi:10.1038/s41467-024-44969-x)
Supplement: Supplementary file 10 — Reporting Summary [file 41467_2024_44969_MOESM10_ESM.pdf]

Corresponding author(s): Jose C. R. Silva

Last updated by author(s): 2023/12/25

Reporting Summary

Nature Portfolio wishes to improve the reproducibility of the work that we publish. This form provides structure for consistency and transparency in reporting. For further information on Nature Portfolio policies, see our Editorial Policies and the Editorial Policy Checklist.

Statistics

For all statistical analyses, confirm that the following items are present in the figure legend, table legend, main text, or Methods section.

n/a	Confirmed	
☐	☒	The exact sample size (*n*) for each experimental group/condition, given as a discrete number and unit of measurement
☐	☒	A statement on whether measurements were taken from distinct samples or whether the same sample was measured repeatedly
☐	☒	The statistical test(s) used AND whether they are one- or two-sided<br>*Only common tests should be described solely by name; describe more complex techniques in the Methods section.*
☒	☐	A description of all covariates tested
☐	☒	A description of any assumptions or corrections, such as tests of normality and adjustment for multiple comparisons
☐	☒	A full description of the statistical parameters including central tendency (e.g. means) or other basic estimates (e.g. regression coefficient) AND variation (e.g. standard deviation) or associated estimates of uncertainty (e.g. confidence intervals)
☐	☒	For null hypothesis testing, the test statistic (e.g. *F*, *t*, *r*) with confidence intervals, effect sizes, degrees of freedom and *P* value noted<br>*Give P values as exact values whenever suitable.*
☒	☐	For Bayesian analysis, information on the choice of priors and Markov chain Monte Carlo settings
☒	☐	For hierarchical and complex designs, identification of the appropriate level for tests and full reporting of outcomes
☒	☐	Estimates of effect sizes (e.g. Cohen's *d*, Pearson's *r*), indicating how they were calculated

Our web collection on statistics for biologists contains articles on many of the points above.

Software and code

Policy information about availability of computer code

Data collection	No software was used for Data Collection.
Data analysis	No custom code was used in this manuscript. List of software used:<br>command line tools: FastQC (v.0.11.2); STAR (v.2.7.9a);<br>R (4.0.5) - Seurat (v.4.1.0); scran (v.1.20.1); batchelor (v.1.8.1); SeuratWrappers (v.0.3.0); DoubletFinder (2.0.3); ggplot2 (3.4.3); pheatmap (1.0.12); GSVA (1.46.0); dplyr (1.1.3); tidyverse (2.0.0)<br>Python (3.8.12) - numpy (1.21.3); scVelo (0.2.5); pandas (1.5.3); matplotlib (3.6.2); scipy (1.9.3); scanpy (1.9.0); anndata (0.8.0)<br>GraphPad Prism (9.5.0), ImageJ (1.53t).

For manuscripts utilizing custom algorithms or software that are central to the research but not yet described in published literature, software must be made available to editors and reviewers. We strongly encourage code deposition in a community repository (e.g. GitHub). See the Nature Portfolio guidelines for submitting code & software for further information.

Data

Policy information about availability of data

All manuscripts must include a data availability statement. This statement should provide the following information, where applicable:

- Accession codes, unique identifiers, or web links for publicly available datasets
- A description of any restrictions on data availability
- For clinical datasets or third party data, please ensure that the statement adheres to our policy

Data availability
RNA-seq datasets generated in this study have been deposited in the Genome Sequence Archive (GSA) in National Genomics Data Center (NGDC) of Chinese Academy of Sciences (CAS) with accession code PRJCA011718 (https://ngdc.cncb.ac.cn/bioproject/browse/PRJCA011718). The raw and processed data are publicly available. Previously published sequencing data that were reanalysed here are available in the GEO or ArrayExpress under the accession codes E-MTAB-3929, GSE136447 and E-MTAB-9388 (scRNA-seq data of E3-E7, D6-D14 and CS7 human embryos); Count matrix of E10-E14 embryo data from Ai et al50 was kindly provided by the authors. GSE134571, GSE156596, GSE171820 and GSE177689 (scRNA-seq data of blastoids reported in previous studies); and the sample GSM2041716, GSM2041717 (UCLA20 hnESCs) and GSM4721334, GSM4721335 (TJ-1# hnESCs). Source data are provided with this paper.

Research involving human participants, their data, or biological material

Policy information about studies with human participants or human data. See also policy information about sex, gender (identity/presentation), and sexual orientation and race, ethnicity and racism.

Reporting on sex and gender	Cell lines of both sexes were used to avoid sex-related biased results. We do not perform or report on any sex-based analysis.
Reporting on race, ethnicity, or other socially relevant groupings	Not applicable.
Population characteristics	Not applicable.
Recruitment	Not applicable.
Ethics oversight	All human ESC, iPSC and blastoid experiments were performed at the Center for Cell Lineage and Atlas (CCLA) of Bioland Laboratory and Guangzhou National Laboratory followed the 2016 and 2021 Guidelines released by the International Society for Stem Cell Research (ISSCR). Human ESC, iPSC and blastoid work was reviewed and approved by the Ethics Committee of Guangzhou National Laboratory.

Note that full information on the approval of the study protocol must also be provided in the manuscript.

Field-specific reporting

Please select the one below that is the best fit for your research. If you are not sure, read the appropriate sections before making your selection.

☒ Life sciences ☐ Behavioural & social sciences ☐ Ecological, evolutionary & environmental sciences

For a reference copy of the document with all sections, see nature.com/documents/nr-reporting-summary-flat.pdf

Life sciences study design

All studies must disclose on these points even when the disclosure is negative.

Sample size	No statistical method was used to predetermine sample size. Generation of spontaneous blastoids depends on the aggregation of stem cells in AggreWell which contain 1200 individual microwells. Individual structures are then recovered for analysis. Given the variation of biological experiments, it is difficult to predict the number of blastoids in each setting. Therefore, instead of predetermining sample size, we ensure all experiments are reproducible between multiple biological replicates and between multiple individual researchers.
Data exclusions	In bulk RNA-seq data analysis, raw reads of fastq format from this study were first processed through in-house perl scripts to obtain clean reads by removing reads containing adapter, reads containing ploy-N and low-quality reads from raw reads. Single-cell RNA-seq data generated in this study was pre-processed through Trimmomatic software to remove adapters and to drop low quality reads or short reads below 26 bps. For data generated in this study, quality controls were performed based on initial evaluation of per-cell quality control metrics. In particular, for the data-sets of spontaneous blastoids cultured in 5iLAF for 0 and 2 days (Smart-Seq2), cells with less than 6000 detected genes or over 15% mitochondrial gene percentage were filtered out; for the data-sets of day 3 and day 6 spontaneous blastoids, the blastoids upon removing MEK/BRAF inhibition as well as the blastoids undergoing post-implantation development (10X Genomics), cells with less than 2500, 4000, 2000 and 2000 detected genes respectively or with total read counts over 100000 or over 25%, 20%, 30% and 25% mitochondrial gene percentage were excluded. DoubletFinder73 software was used to detect and filter hybrid cells. In later analysis of the data-set of day 6 spontaneous blastoids, a 'doublet' cluster was detected and excluded. For public data, cells with less than 2000 detected genes or >= 20% mitochondrial gene percentage were filtered out. Genes detected in at least 3 cells were retained.

Replication	All experiments were repeated independently across individual frozen cell stocks of different passages. We have also used 3 cell lines (two ESC lines TJ#1 ES and H9, an iPSC line STiPS O-XX1, to repeat our experiments. We have also converted naive hPSCs by different methods and repeated our experiments across the newly generated cell lines. We ensure all experiments are reproducible between multiple biological replicates and between multiple individual researchers.
Randomization	For experiments where blastoids were allocated into different groups (different media or different strategies), the indicated medium is directly added to the AggreWell which contains 1200 microwells. For efficiency calculation and differentiation experiments, the initial cells are randomly allocated into the dish. For spontaneous blastoid single-cell sequencing and post-implantation development experiments, only blastoids with a blastocyst-like structure are selected.
Blinding	Investigators were not blinded to the experimental groups. It was crucial to keep good and consistent status of the cell lines, therefore, it was not possible to blind the investigators during experiments and data analysis.

Reporting for specific materials, systems and methods

We require information from authors about some types of materials, experimental systems and methods used in many studies. Here, indicate whether each material, system or method listed is relevant to your study. If you are not sure if a list item applies to your research, read the appropriate section before selecting a response.

Materials & experimental systems

n/a	Involved in the study
☐	☒ Antibodies
☐	☒ Eukaryotic cell lines
☒	☐ Palaeontology and archaeology
☒	☐ Animals and other organisms
☒	☐ Clinical data
☒	☐ Dual use research of concern
☒	☐ Plants

Methods

n/a	Involved in the study
☒	☐ ChIP-seq
☒	☐ Flow cytometry
☒	☐ MRI-based neuroimaging

Antibodies

Antibodies used	Sox2 (Invitrogen, 14-9811-80, Clone Btjce, 1:200); Gata6 (R&D systems, AF1700, 1:40); Gata3 (Abcam, ab199428, Clone EPR16651, 1:250); ZO-1 (Invitrogen, 339100, Clone ZO1-1A12, 1:100); Nanog (Cell Signaling Technology, 4893, 1:1000); Krt18 (Sigma, SAB4501665, 1:200); Oct4 (Abcam, ab19857, 1:200); Tp63 (R&D systems, AF1916, 1:40); Cdx2 (Emergo Europe, MU392A-5UC, Clone CDX2-88, 1:50); Pdgfra (Cell Signaling Technology, 3174, 1:1000); Gata4 (Cell Signaling Technology, 36966, 1:400); Gata4 (Invitrogen, 14-9980-82, 1:100); Sox17 (R&D systems, AF1924, 1:100); Cer1 (R&D systems, AF1075, 1:200); Brachyury/T (R&D systems, AF2085, 1:200); CGB (Abcam, ab53087, 1:250); HLA-G (Abcam, ab7759, Clone MEM-G/1, 1:100).<br>Alexa fluor 488 donkey anti-mouse IgG (Invitrogen, A21202, 1:1000); Alexa fluor 555 donkey anti-mouse IgG (Invitrogen, A31570, 1:1000); Alexa fluor 647 donkey anti-mouse IgG (Invitrogen, A31571, 1:1000); Donkey anti-Rabbit IgG (H+L) Highly Cross-Adsorbed Secondary Antibody, Alexa Fluor 488 (Invitrogen, A21206, 1:1000); Donkey anti-Rabbit IgG (H+L) Highly Cross-Adsorbed Secondary Antibody, Alexa Fluor 555 (Invitrogen, A31572, 1:1000); Donkey anti-Rabbit IgG (H+L) Highly Cross-Adsorbed Secondary Antibody, Alexa Fluor 647 (Invitrogen, A31573, 1:1000); Donkey anti-Rat IgG (H+L) Highly Cross-Adsorbed Secondary Antibody, Alexa Fluor 488 Invitrogen, A21208, 1:1000); Donkey anti-Rat IgG (H+L) Highly Cross-Adsorbed Secondary Antibody, Alexa Fluor 594 (Invitrogen, A21209, 1:1000); Chicken anti-Rat IgG (H+L) Cross-Adsorbed Secondary Antibody, Alexa Fluor 647 (Invitrogen, A21472, 1:1000); Donkey anti-Goat IgG (H+L) Cross-Adsorbed Secondary Antibody, Alexa Fluor 488 (Invitrogen, A11055, 1:1000); Donkey anti-Goat IgG (H+L) Cross-Adsorbed Secondary Antibody, Alexa Fluor 594 (Invitrogen, A11058, 1:1000); polyclonal Secondary Antibody to Goat IgG - H&L (Alexa Fluor® 647),pre-adsorbed (abcam, ab150135, 1:1000)
Validation	All primary antibodies are validated for detection of the human antigen of interest according to manufacturer's websites.

Eukaryotic cell lines

Policy information about cell lines and Sex and Gender in Research

Cell line source(s)	TJ 1# ES is provided from Dr. Shaorong Gao and Dr.Yixuan Wang in Tongji University. TiPS O-XX1 cell line is provided from Dr.Wenjuan Li and Dr.Miguel A. Esteban in Guangzhou Institutes of Biomedicine and Health. H9 line is conserved under Dr.Jose C. R. Silva in Guangzhou Medical University and Guangzhou National Laboratory.
Authentication	Karyotype and sequencing data confirmed expected sex and karyotype, gene reporters and cell identity via SNPs.
Mycoplasma contamination	All cell lines were regularly tested and were ensured mycoplasma-free.
Commonly misidentified lines (See ICLAC register)	Not applicable.

Plants

Seed stocks	Not applicable.
Novel plant genotypes	Not applicable.
Authentication	Not applicable.
